# Unraveling the enigma of tumor-associated macrophages: challenges, innovations, and the path to therapeutic breakthroughs

**DOI:** 10.3389/fimmu.2023.1295684

**Published:** 2023-11-14

**Authors:** Shengwen Shao, Huilai Miao, Wenxue Ma

**Affiliations:** ^1^ Clinical Research Center, The Second Affiliated Hospital of Guangdong Medical University, Zhanjiang, Guangdong, China; ^2^ Department of Hepatobiliary Surgery, The Second Affiliated Hospital of Guangdong Medical University, Zhanjiang, Guangdong, China; ^3^ Department of Hepatobiliary Surgery, Liaobu Hospital of Dongguan City, Dongguan, Guangdong, China; ^4^ Department of Medicine, Moores Cancer Center, and Sanford Stem Cell Institute, University of California, San Diego, La Jolla, CA, United States

**Keywords:** tumor-associated macrophages (TAM), tumor microenvironment (TME), phenotypic diversity, regulatory signaling pathways, clinical trials, cancer immunotherapy

## Abstract

Tumor-associated macrophages (TAMs) are integral to the tumor microenvironment (TME), influencing cancer progression significantly. Attracted by cancer cell signals, TAMs exhibit unparalleled adaptability, aligning with the dynamic tumor milieu. Their roles span from promoting tumor growth and angiogenesis to modulating metastasis. While substantial research has explored the fundamentals of TAMs, comprehending their adaptive behavior, and leveraging it for novel treatments remains challenging. This review delves into TAM polarization, metabolic shifts, and the complex orchestration of cytokines and chemokines determining their functions. We highlight the complexities of TAM-targeted research focusing on their adaptability and potential variability in therapeutic outcomes. Moreover, we discuss the synergy of integrating TAM-focused strategies with established cancer treatments, such as chemotherapy, and immunotherapy. Emphasis is laid on pioneering methods like TAM reprogramming for cancer immunotherapy and the adoption of single-cell technologies for precision intervention. This synthesis seeks to shed light on TAMs’ multifaceted roles in cancer, pinpointing prospective pathways for transformative research and enhancing therapeutic modalities in oncology.

## 1 Introduction

Tumor-associated macrophages (TAMs) are integral immune cells that occupy a pivotal position within the tumor microenvironment (TME). With their dual roles in both promoting and inhibiting tumor activities, they stand at the forefront of cancer progression research (1–5). Rather than mere bystanders, their active presence in the TME is influenced predominantly by signals from cancer cells, leading to their active recruitment (6, 7).

Numerous preclinical and clinical studies have highlighted the promising therapeutic implications of targeting TAMs. However, this journey presents its set of challenges, from understanding TAM subtypes to crafting precise therapies (8–11). The inherent adaptability of TAMs to diverse stimuli brings light to the unpredictable nature of therapeutic outcomes, further complicating the therapeutic landscape (12, 13).

This review has multifaceted objectives. We aim to deepen the understanding of both researchers and clinicians about TAMs, spotlighting their intricate roles in the complex landscape of cancer progression and potential interventions. We delve into aspects like TAM polarization, the influence of cytokines and chemokines, and related metabolic pathways. Furthermore, we investigate the potential of merging TAM-targeted strategies with conventional treatments, covering chemotherapy, radiation, and immunotherapy. This includes the innovative concept of utilizing reprogrammed TAMs for cancer immunotherapy (14) and employing single-cell-based technologies for personalized therapeutic interventions (15).

In summary, this review highlights the diverse roles of TAMs in oncology, offering a landscape teeming with both opportunities and complexities. We underscore the urgency to refine TAM-focused therapeutic strategies and shed light on ongoing advancements and challenges in cancer immunotherapy. Our intent is to pave a lucid path ahead, pinpointing areas warranting thorough exploration. In presenting this consolidated overview, we aim to equip our readers, whether researchers or medical professionals, with a comprehensive understanding of TAMs in the broader context of cancer progression and therapeutic intervention.

## 2 The multifaced role of TAMs in the TME

TAMs are essential components of the TME across various cancers. Originating from circulating monocytes, they migrate to the tumor site, these monocytes differentiate into macrophages (MΦ), which are TAMs under the influence of signals from both distressed tissues and neoplastic cells. As these macrophages immerse themselves within the tumor milieu, they further mature into the TAMs (6, 7). Due to their vast phenotypic diversity, TAMs possess the ability to transition between pro-tumor (M2) and anti-tumor (M1) roles, influenced by cues from the TME. Depending on their state and the TME’s signaling, TAMs might either bolster or hinder tumor progression (1, 16).

### 2.1 Phenotypic diversity and TAM interaction within the TME

TAMs are central players within the TME, characterized by a broad spectrum of phenotypic attributes. Their inherent phenotypic diversity equips them to assume varied roles, ranging from promoting to counteracting tumor activities (1, 4, 17, 18). This diversity becomes particularly pronounced when observing TAM behavior across different cancer types. For instance, in breast cancer, TAMs often support tumor growth by promoting angiogenesis (19) through releasing growth factors such as vascular endothelial growth factor (VEGF), transforming growth factor-β (TGF-β), and platelet-derived growth factor (PDGF) (20), while in melanoma, they predominantly exhibit immune-suppressive characteristics (21, 22).

Diving deeper into the specific factors released by TAMs, these cells secrete a variety of cytokines and growth factors that drive oncogenic activities (17, 23). Notably, the release of cytokines such as IL-6 and IL-10 promotes an immunosuppressive TME, facilitating tumor escape from immune surveillance (24, 25). On the angiogenic front, aside from the well-documented VEGF, TAMs release additional pro-angiogenic factors that augment tumor blood supply, enhancing nutrient availability for rapidly growing tumors (26–28). The interactions between these cytokines and growth factors not only fuel tumor growth and invasion but also shape the overall dynamics of the TME, reinforcing the pro-tumorigenic nature of TAMs (29, 30).

A deeper dive into the molecular mechanisms reveals that TAMs, while often manipulated by tumors to support their growth, can also be utilized in novel therapeutic strategies. There’s an ongoing shift in the therapeutic paradigm to not simply eliminate TAMs but to modulate their behavior. By targeting specific signaling pathways and employing checkpoint inhibitors, there’s potential to reprogram pro-tumorigenic M2-like TAMs into tumor-inhibiting M1-like counterparts (10, 31, 32). Such an approach aims to harness the inherent capabilities of TAMs in tissue repair and homeostasis, converting them from potential adversaries to allies in cancer therapy.

The differentiation into specific phenotypes is largely steered by the cytokines present in the TME, acting as molecular directives for TAMs. M1-type macrophages, which are pro-inflammatory, instigate immune responses against tumor cells and potentially restrict tumor expansion. Conversely, M2-type macrophages, which exhibit anti-inflammatory tendencies, generally facilitate tissue repair and boost angiogenesis. However, in the TME, they can inadvertently foster tumor progression and aid metastasis (33, 34).

TAMs’ presence in the TME isn’t solitary; they are in constant dialogue with other elements like cancer cells, stromal cells, and the extracellular matrix. Through these interactions, TAMs can support angiogenesis, helping form new blood vessels to feed the tumor, but also hinder immune responses, further aiding tumor cells in metastasizing to new locations (1, 31, 32).

### 2.2 Recruitment, maturation, and regulatory signaling pathways

The TME, serves as a hub of complex interactions, with its cellular and non-cellular components guiding the recruitment and maturation of TAMs. Molecular signals from tumor and stromal cells, notably the chemokine CCL2, actively attract circulating monocytes to the tumor vicinity. Upon arrival, monocytes are further influenced by diverse signals. Growth factors, prominently macrophage-colony stimulating factor (CSF-1) emanating from tumor cells, hypoxia-responsive elements like hypoxia-inducible factor 1-alpha (HIF-1α), and metabolic derivatives such as lactate play vital roles. The significance of contact-dependent signaling, exemplified by the CD200 and CD200R interaction, is also noteworthy. These myriad of cues steer monocytes to differentiate into macrophages, which further evolve into TAMs, reflecting the deep symbiosis between neoplastic cells and TAM adaptability. Such understanding hints at potential therapeutic interventions (6, 7).

In the realm of signaling pathways dictating TAM recruitment and maturation, the CSF1/CSF1R and CCL2/CCR2 axes stand out. The CSF1/CSF1R pathway is indispensable for macrophage survival and maturation, while the CCL2/CCR2 axis chiefly orchestrates monocyte trafficking to the TME (20, 33). Venturing beyond these, other pivotal pathways like STAT3, NF-κB, and PI3K are instrumental in shaping TAM activities, steering them toward either tumor promotion or suppression (35–37).

Given the central role of transcription factors, especially STAT3 and NF-κB, in TAM signaling, it’s pertinent to understand their modulators. Tyrosine kinase inhibitors (TKIs), such as Sunitinib and Sorafenib, have been identified to target the CSF1/CSF1R (38) and STAT3 (36, 39) pathways, respectively, implying potential avenues to redirect TAM functions. Additionally, small molecular inhibitors like BAY 11-7082, targeting the NF-κB pathway, offer promising leads. A deeper comprehension of these inhibitors can elucidate opportunities to alter TAM behavior favorably, rendering enhanced prognoses for patients.

Harnessing this intricate molecular knowledge paves the way for innovative therapeutic strategies, emphasizing the recalibration of TAM dynamics within the TME.

### 2.3 Modulation of TAM roles by tumor attributes

TAMs are pivotal constituents of the TME. Their behavior and roles are profoundly influenced by the broader tumor context. Key tumor attributes, including its stage and anatomical location, play pivotal roles in determining TAM behavior (40).

In the early stage of tumor development, the TME is characterized by a dominant immune response. This is primarily facilitated by M1-type TAMs known for their pro-inflammatory and anti-tumor properties. Their abundance in these early stages suggests the body’s proactive defense against tumor formation, which could be indicative of positive outcomes for patients (41).

As the tumor evolves, a significant shift occurs within the TME. M2-type TAMs, which assist tumor growth through angiogenesis, immune suppression, and tissue reconstruction, become more prevalent (4, 42). Their increased presence in the advanced stages reflects the tumor’s adeptness at modulating its surrounding environment. Such adaptability might enhance the tumor’s resilience against therapeutic strategies (31, 43–45).

Recognizing the dynamic roles of TAMs, influenced by tumor attributes, underscores the need for an in-depth understanding of the tumor’s condition. Grasping these changes can inform and refine therapeutic approaches, potentially enhancing treatment efficacy and overall patient prognosis.

## 3 Recognizing the limitations in TAM research

In the pursuit of understanding the role of TAMs in tumor biology, it’s imperative to address the inherent challenges and discrepancies that stand as obstacles to a thorough comprehension and its subsequent clinical application.

### 3.1 Challenges in TAM isolation techniques

TAMs, integral to the TME, play a pivotal role in influencing tumor dynamics and determining therapeutic responses (13, 46). Successfully isolating TAMs is essential for understanding their functions and devising effective therapeutic strategies (4, 47). However, each isolation technique presents distinct challenges.

For instance, while enzymatic digestion is adept at dismantling the extracellular matrix (ECM), its over-reliance on specific antigenic markers can lead to inaccurate TAM representation (47). Techniques like magnetic associated cell sorting (MACS) & fluorescence-activated cell sorting (FACS), though efficient, can be plagued by inconsistencies stemming from marker variability (48–51).

Adhesion-based techniques, tapping into TAMs’ natural propensity to adhere to certain surfaces, aren’t without their pitfalls. The adherence properties of TAMs, shaped by their originating tumor microenvironments, can vary. For example, TAMs from breast tumors may adhere differently than those from lung tumors, leading to disparities in isolation outcomes. Such variations underscore the influence of unique tumor microenvironments on TAM cellular behaviors, including adhesion (52, 53).

Other methods like density gradient centrifugation might produce heterogeneous cell populations, warranting further purification (54). While laser capture microdissection (LCM) offers precision, its stringent requirements could limit its widespread application (46, 55). Furthermore, tissue microarrays, despite their potential insight, might overlook specific TAM subsets depending on the selected markers (56).

### 3.2 Challenges in transitioning research to clinical settings

In various cancers, TAMs exhibit distinct interactions and behaviors shaped by the specific TME. For example, in breast cancer, TAMs often support tumor growth, and metastasis, and hinder anti-tumor immune responses (19, 20). In contrast, cervical cancer showcases a strong correlation between heightened TAM infiltration and advanced disease stages, with TAMs enhancing angiogenesis and suppressing immune reactions against cancer cells (57, 58). In melanoma, TAMs not only aid tumor progression but also sometimes foster resistance to standard treatments, although emerging therapies targeting TAMs offer hope (59, 60). Particularly in non-small cell lung cancer, a subtype of lung cancer, the presence of TAMs relates to tumor progression, metastasis, and even resistance to certain treatments, often indicating a reduced survival rate for affected patients (61, 62).

Transitioning these insights from laboratory studies to clinical applications is fraught with challenges. Central to this transition is the need to perfect TAM isolation methods suitable for clinical environments. Embracing and advocating for innovative, streamlined techniques will be critical, aiming to provide a consistent foundation for TAM research and thereby deepening our knowledge while fast-tracking TAM-centric therapeutic avenues.

## 4 TAM interactions and polarization dynamics

The intricate relationship between TAMs and the TME is a complex interweaving of factors, ranging from TAM activation dynamics, and polarization predispositions, to the distinctive features of the TME, governed by cancer type and its progression stage (17). Understanding this multifaceted interplay is essential to develop efficient TAM-targeted immunotherapies.

### 4.1 TAM regulatory mechanisms

Within the TME, the behavior and function of TAMs are influenced by multifarious factors including cytokines, metabolic cues, ECM components, and, crucially, hypoxia (63, 64). Hypoxia, characterized by reduced oxygen availability, is a hallmark in the TME and serves as a pivotal modulator of TAM polarization. In response to hypoxic conditions, TAMs are driven toward the M2 phenotype, which tends to support tumor growth and metastasis (65, 66). This hypoxia-induced M2 polarization activates specific transcriptional profiles that are critical for the cellular communication between TAMs and the tumor’s stromal components, including fibroblasts, endothelial cells, and other immune cells (67).

Additionally, TAMs display distinct metabolic signatures which further modulate their activities within the TME (68). Elevated glycolysis paired with decreased oxidative phosphorylation is often observed in TAMs, facilitating their survival in the nutrient depleted and hypoxic TME (68, 69). Increased fatty acid synthesis and uptake are also characteristic of TAMs and are correlated with their immunosuppressive functionalities (68, 70, 71). These unique metabolic patterns significantly contribute to angiogenesis, anti-tumor immunity suppression, and the facilitation of tumor cell invasion and metastasis.

Moreover, the molecular underpinnings governing TAM polarization are intricate (72, 73). When exposed to Th1 cytokines like IFN-γ, TAMs undergo a shift toward the M1 phenotype via the JAK-STAT1 pathway, augmenting their tumoricidal capabilities (74, 75). Lipopolysaccharide (LPS), an endotoxin predominantly derived from Gram-negative bacteria within the tumor microenvironment, serves as an influential modulator for TAMs. It interacts with TLR4 to strengthen the M1-type TAM response via the NF-κB and MAPK pathways (76–78). The source of this LPS can be multifaceted, stemming from tumor-associated bacteria, systemic sources, or even mimics from necrotic tumor cells within the TME (79, 80). Recent evidence suggests that certain tumors, especially in organs with a rich microbial environment, possess an associated microbiome. The gram-negative bacteria present within these tumors release LPS, which becomes a pivotal component of the TME, affecting the behavior of various immune cells, including TAMs. On the contrary, Th2 cytokines, notably IL-4 and IL-13, prompt a tilt toward the M2 phenotype through the activation of the JAK-STAT6 signaling pathway (81). This shift, in turn, promotes tissue repair, angiogenesis, and tumor progression (61). Other molecules such as TGFβ, chemokines, and PGE2 further emphasize the pro-tumorigenic M2 profile (44, 82–84).

Unraveling these multifaceted hypoxia-driven interactions, combined with the intricate molecular mechanisms governing TAM polarization, offers promising avenues for therapeutic interventions (85).

### 4.2 TAM-TME interaction dynamics

The hypoxic environment within the TME has profound implications for the behavior and functionality of TAM. Hypoxia not only drives TAM polarization but also modulates the complex interplay between TAMs and other cellular components of the TME, including immune cells, stromal cells, and cancer cells (29, 86). This low-oxygen condition intensifies the interactions between TAMs and other TME cells emphasizing the pivotal roles TAMs play, for instance, regulating T-cell functions (87, 88). Additionally, hypoxia augments TAM-associated processes like angiogenesis and communication with stromal cells, ultimately fostering tumor growth (89–91).

Within the TME, several cell types actively modify the microenvironment, each contributing distinct molecular mechanisms that collectively influence tumor dynamics (92). TAMs play a pivotal role by releasing various cytokines and growth factors, inducing angiogenesis via VEGF secretion, fostering tissue remodeling with MMPs, and suppressing immune responses through factors like IL-10 and TGF-β (4, 18, 32, 35). Complementing this, Cancer-Associated Fibroblasts (CAFs) actively remodel the extracellular matrix, heightening tumor stiffness that augments cancer cell migration (93). These fibroblasts secrete growth factors, notably TGF-β, propelling cancer cell proliferation and facilitating epithelial-mesenchymal transition (EMT) (94). CAFs also contribute to the immunosuppressive nature of the TME, recruiting regulatory T cells (Tregs) via CCL2 secretion (83). Similarly, Myeloid-Derived Suppressor Cells (MDSCs) are essential players in dampening the anti-tumor immune response, inhibiting T cell activation with reactive species, and depleting crucial amino acids, which further impede T cell functionality (95).

Macrophages are classically polarized into two primary phenotypes: M1, known for its pro-inflammatory attributes, and M2, recognized for its anti-inflammatory characteristics (74). The M1 macrophages predominantly defend against pathogens and participate in early wound healing, while M2 macrophages orchestrate tissue repair, immunoregulation, and inflammation resolution (96).

Diving deeper, M2 macrophages are further segmented into subtypes, namely M2a, M2b, M2c, and M2d, each distinguished by their specific roles, stimuli responsiveness, and cytokine production (97). Particularly, the M2b subtype, emerging as the focal point of numerous studies, exhibits hybrid pro- and anti-inflammatory activities, asserting their significance in varied physiological and pathological contexts (98).

Current research accentuates the diverse roles of M2b macrophages within both oncological and infectious contexts. M2b macrophages not only participate in cancer promotion, metastasis, and recurrence but also play crucial roles during infectious challenges. These cells, unique among macrophages, are characterized by their secretion of specific pro-inflammatory cytokines. Given their capability to accentuate infections while concomitantly suppressing certain immune responses, there is an increasing emphasis on understanding M2b polarization dynamics. This focus arises from the potential of targeting M2b macrophages for therapeutic innovations, both in cancer and infectious disease scenarios (99–101).

M2b macrophages can be identified based on their distinct cytokine secretion patterns. They are known to express high levels of the anti-inflammatory cytokine IL-10. At the same time, they exhibit reduced IL-12 levels. These cells also possess the capability to release significant quantities of pro-inflammatory agents, such as IL-1β, IL-6, and TNF-α. Interestingly, despite this pro-inflammatory profile, M2b macrophages maintain high IL-10 levels, demonstrating their multifaceted immunomodulatory roles (22). The following **Table 1** elucidates the determinants guiding the polarization of macrophages into either M1 or M2 TAM phenotypes.

**Table 1:** Determinants of circulating macrophage polarization into M1 or M2 TAMs (22, 31, 32, 99–102).

MΦ Types	M1 MΦ	M2 MΦ			
		M2a MΦ	M2b MΦ	M2c MΦ	M2d MΦ
Stimuli	IFN-γ and/or LPS	IL-4 and/or IL-13	immune complexes and/or TLR agonists	IL-10 and/or TGF-β	TLR agonists, adenosine, tumor-associated factors
Function	Pro-inflammatory	Anti-inflammatory	Immunoregulatory (Pro- and Anti-inflammatory)	Anti-inflammatory	Pro-angiogenic
Cytokine profile	Produce IL-12, IL-23, and iNOS	Produce IL-10 and arginase 1	Produce IL-1β, IL-6, TNF-α, and high IL-10, low IL-2	Produce IL-10 and TGF-β	Produce IL-4, IL-10, and TGF-β
Cell response	Promote Th1 cell response	Promote Th2 cell response	Promote Th2 (low induction of Th1) cell response	Promote Th2 cell response	Promote Th2 cell response
Function	Inhibit tumor growth	Promote tumor growth	Promote tumor growth & infection due to blunting of immune response	Promote angiogenesis	Promote tumor growth
Chemokine profile	CCL3, CCL4, CXCL9, CXCL10, CXCL11	CCL17, CCL18, CCL22, CCL24			
Surface markers	CD80, CD86, MHC II	CD206, CD163, MRC1, IL-10 receptor			

### 4.3 TAM polarization in the TME

Hypoxia in the TME is a pivotal determinant in guiding TAM polarization. Under these low-oxygen conditions, CD8^+^ T cells lean toward M1-type polarization, whereas Tregs predominantly skew toward the M2-type (9, 27, 102, 103). The interplay between immune checkpoints, especially those involving PD-L1 and PD-1, further intricates TAM polarization within hypoxic TME. he implications of this interaction might have substantial consequences for tumor progression and its subsequent therapeutic targeting (104, 105).

Inflammatory cytokines, such as IL-6, TNF-α, and IL-1β, are instrumental in determining TAM behavior within the TME (106). These cytokines actively guide the polarization of macrophages toward an M2-like, tumor-promoting phenotype. For instance, IL-6, by activating the STAT3 signaling pathway, fosters M2 macrophage differentiation, which in turn is associated with activities that promote tumor growth and progression.

In addition to cytokines, ECM components present in the TME, notably hyaluronan and collagen, play significant roles in TAM functionality. Hyaluronan interacts with TAM receptors like CD44, triggering intracellular pathways that enhance the pro-tumorigenic functions. In contrast, collagen provides a structural scaffold that assists TAM. When collagen binds to integrin receptors on TAMs, it influences their differentiation and function within the TME (32, 107, 108).

**Figure 1:**
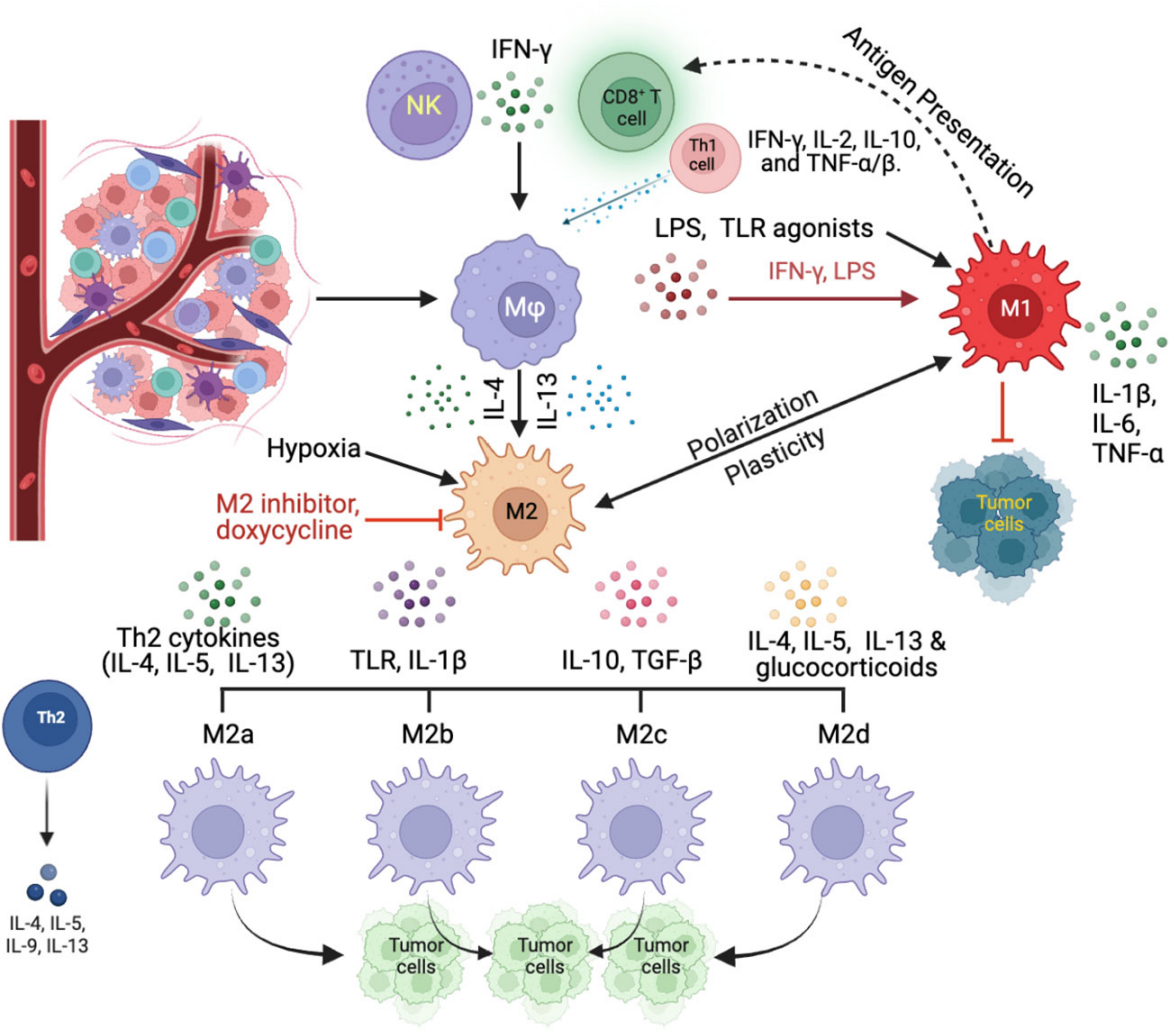
Dynamics of TAM polarization within the TME. TAMs, originating from circulating monocytes, differentiate into macrophages within the TME in response to specific signals. Key influencers include the cytokine IFN-γ, sourced from activated CD8^+^ T cells and NK cells, as well as a combination of IFN-γ, IL-2, IL-10, and TNF-α/β from Th1 cells (CD4^+^ T cells). External factors like LPS, predominantly derived from Gram-negative bacteria within the tumor microenvironment, and other TLR agonists, which are typically released from bacteria, and necrotic tumor cells, or systemic sources, favor the development of the M1 macrophage phenotype. In contrast, potent Th2 cytokines, such as IL-4 and IL-13, along with hypoxic conditions, steer macrophages toward the TAM2 direction. TAM1 macrophages, influenced dominantly by IFN-γ and LPS, assume a pro-inflammatory stance, producing cytokines like IL-1β, IL-6, and TNF-α that can promote tumor cell apoptosis. On the other hand, TAM2 macrophages, shaped by IL-4 and IL-13, exhibit an anti-inflammatory profile, secreting cytokines such as IL-10 and TGF-β, which not only suppress immune responses but also enhance angiogenesis processes.

A thorough comprehension of the interplay and dynamics of TAM in a hypoxic TME is essential for devising therapeutic strategies that amplify anti-tumor responses. **Figure 1** illustrates the dynamics of TAM polarization within the TME, providing a visual representation of the factors that influence their differentiation and subsequent roles in tumor progression and immune response modulation.

## 5 Ongoing clinical trials

TAMs play a pivotal role in tumor progression and therapeutic outcomes. Given this, there is significant interest in the oncological domain regarding TAMs, with numerous clinical trials emphasizing TAM-centered therapies.

### 5.1 Clinical investigations centered around TAMs

TAMs have emerged as key players in the TME, guiding both tumor progression mechanisms and innovative therapeutic strategies. Several clinical trials are underway to harness the potential of TAMs, and we have sourced these from the extensive database www.clinicaltrials.gov. **Table 2** provides a snapshot of these trials, both ongoing and completed.

**Table 2:** Clinical trials involving TAMs in cancers (www.clinicaltrials.gov).

NCT Number	Phases	Study title	Conditions	Completion Date
NCT00690261	N/A	The Impact of M1/M2 TAM Polarization on Cancer Progression and Prognosis Prediction	Tumor, Lung Cancer	August 2010
NCT01551251	N/A	Tumor-Associated Macrophage in Advanced Non-small Cell Lung Cancer	Advanced Non-small Cell Lung Cancer	December 2010
NCT05053750	Early Phase 1	A Pilot Study of Weekly Paclitaxel, Bevacizumab, and Tumor-Associated Macrophage Targeted Therapy (Zoledronic Acid) in Women with Recurrent, Platinum-resistant, Epithelial Ovarian, Fallopian Tube or Primary Peritoneal Cancer	Epithelial Ovarian, Fallopian Tube, Primary Peritoneal Cancer	March 31 2023
NCT01770353	Phase 1	MM-398 (Nanoliposomal Irinotecan, Nal-IRI) to Determine Tumor Drug Levels and to Evaluate the Feasibility of Ferumoxytol Magnetic Resonance Imaging to Measure Tumor-Associated Macrophages and to Predict Patient Response to Treatment	Solid Tumors, ER/PR Positive Breast Cancer, Triple Negative Breast Cancer, Metastatic Breast Cancer, Metastatic Breast Cancer with Active Brain Metastasis	Oct. 2 2018
NCT03888638	N/A	The Role of Tumor-associated Macrophages in Colorectal Liver Metastases	Colorectal Liver Metastases, Colorectal Cancer, Liver Metastases, Immunotherapy	March 1 2019
NCT01493817	N/A	Biomarkers in Samples from Younger Patients with Wilms Tumor	Wilms Tumor and Other Childhood Kidney Tumors	Completed
NCT02472275	Phase 1	PLX3397, Radiation Therapy, and Antihormone Therapy in Treating Patients with Intermediate- or High-Risk Prostate Cancer	Stage I Prostate Adenocarcinoma, Stage II Prostate Adenocarcinoma, Stage III Prostate Adenocarcinoma	August 5 2019
NCT04776980	Early Phase 1	Multimodality MRI and Liquid Biopsy in GBM	Multiforme Glioblastoma, Brain Tumor, Adult Glioblastoma, Recurrent Brain Tumor, Primary Brain Tumor	June 22 2022
NCT04168528	Phase 1, Phase 2	Phase I/IIa Study of 68GaNOTA-Anti-MMR-VHH2 for PET/CT	Malignant Solid Tumor, Breast Cancer, Head and Neck Cancer, Melanoma (Skin)	April 2023
NCT04663126	Early Phase 1	Feasibility of IV Tc-99m-tilmanocept for Imaging of M2-type TAMs in Metastatic Melanoma	Melanoma	December 2022
NCT03397238	N/A	Myeloid Cell Reprogramming in Thyroid Carcinoma	Thyroid Cancer	January 2021
NCT01316822	Phase 1	A Study of ARRY-382 in Patients with Selected Advanced or Metastatic Cancers	Metastatic Cancer	October 2012
NCT00979277	N/A	Transcriptional and Molecular Characterization of Tumor-Associated Monocytes/Macrophages in Human Cancers	Tumor, Cancer	Unknown

Among these trials of NCT04776980 and 687 NCT04168528, the former employs advanced imaging and biopsy methods to study TAMs within the glioblastoma multiforme (GBM) tumor environment. The latter focuses on a radiolabeled agent targeting the MMR (macrophage mannose receptor), predominantly found on M2-polarized TAMs, allowing researchers to visualize the TAM distribution in various malignancies. Another notable trial, NCT01316822, assesses ARRY-382, a targeted inhibitor against the CSF1/CSF1R pathway essential for TAM activity and differentiation. The emphasis on TAMs in these trials showcases their increasing importance in oncology, with potential applications in cancer treatment and patient care.

### 5.2 The potential of CSF1R as a therapeutic target

The Colony-stimulating factor 1 receptor (CSF1R) is fast gaining traction in the realm of cancer therapeutics. This receptor tyrosine kinase is a pivotal element found in TAMs. It plays an indispensable role in orchestrating the growth and survival of TAMs making it a compelling therapeutic target (109, 110).

The therapeutic potential of agents that inhibit CSF1R is a topic of considerable interest and is currently being scrutinized in several clinical trials. One standout Phase 2 clinical has been focusing on gauging the collective effectiveness of the CSF1R inhibitor, namely pexidartinib in tandem with pembrolizumab for treating patients with advanced melanoma cases (10, 111–113).

Currently, a commendable number of 20 clinical trials are actively in progress. These trials are delving into the potential implications of CSF1R in the field of Oncology. A detailed breakdown and specifics of these trials can be perused in **Table 3**.

**Table 3:** Clinical trials involving CSF1R as a therapeutic target in cancers (www.clinicaltrials.gov).

NCT Number	Phases	Study Title	Conditions	Completion Date
NCT04648254	Phase 1	Oral Axl/Mer/CSF1R Selective Tyrosine Kinase Inhibitor in Patients with Advanced Solid Tumor	Solid Tumor, Advanced Cancer, Metastatic Cancer	11/18/2023
NCT05438420	Phase 1/Phase 2	Oral Axl/Mer/CSF1R Selective Tyrosine Kinase Inhibitor Q702 in Combination with Pembrolizumab in Patients with Selected Advanced Solid	Esophageal Cancer, Gastric Cancer, Hepatocellular Cancer, Cervical Cancer	6/30/2026
NCT05438420	Phase 1/Phase 2	Study of NMS-03592088 in Patients with Relapsed or Refractory AML or CMML	Acute Myeloid Leukemia (AML), Chronic Myelomonocytic Leukemia (CMML)	9/30/2023
NCT03993873	Phase 1/Phase 2	Study of TPX-0022 in Patients with Advanced NSCLC, Gastric Cancer, or Solid Tumors Harboring Genetic Alterations in MET	Advanced Solid Tumor, Metastatic Solid Tumors, MET Gene Alterations	11/1/2023
NCT04848116	Phase 2	Neoadjuvant Targeting of Myeloid Cell Populations in Combination with Nivolumab in Head & Neck Ca	Head and Neck Squamous Cell Carcinoma	4/1/2026
NCT05020743	Phase 1/Phase 2	Study of DCC-3014 in Patients with Advanced Tumors and Tenosynovial Giant Cell Tumor	Advanced Malignant Neoplasm, Pigmented Villonodular Synovitis, Giant Cell Tumor of Tendon Sheath, Tenosynovial Giant Cell	6/1/2024
NCT05020743	Phase 1	Phase Ib/II Study of Chiauranib in Patients with Small Cell Lung Cancer	Small Cell Lung Cancer	12/30/2022
NCT05494580	Phase 1/Phase 2	Pamiparib Plus Surufatinib in Patients with Platinum-resistant Ovarian Cancer	Ovarian Cancer, Platinum-resistant Ovarian Cancer, Fallopian Tube Carcinosarcoma, Primary Peritoneal Cancer	8/10/2025
NCT05627427	Phase 2	Multi-cohort Study of Surufatinib Plus Sintilimab in Metastatic NEN and Pancreatic Carcinoma Who Failed Standard Chemotherapy	Neuroendocrine Tumor Grade 3, Neuroendocrine Carcinoma, Pancreatic Carcinoma	12/31/2024
NCT05627427	N/A	Mass Balance Study of [14C] Chiauranib	Small Cell Lung Cancer (SCLC)	6/30/2023
NCT04830813	Phase 3	Phase 3 Clinical Study of Chiauranib Capsule in Patients with Small-cell Lung Cancer	Small Cell Lung Cancer (SCLC)	12/31/2024
NCT05273099	N/A	Molecular Biomarkers Predicting Early Development of Endometrial Carcinoma	Cancer of Endometrium	12/1/2023
NCT04622865	Phase 2	APUR: Testing the Use of FDA Approved Drugs That Target a Specific Abnormality in a Tumor Gene in People with Advanced Stage Cancer	Non-Hodgkin Lymphoma, Multiple Myeloma, Advanced Solid Tumors	12/31/2025
NCT04622865	Phase 2	Biomarker Driven Trial of VEGFR2 Inhibitor in Advanced Sarcoma	Sarcoma	8/25/2023
NCT02171104	Phase 2	A Study Evaluating the Activity of Anti-cancer Treatments Targeting Tumor Molecular Alterations/Characteristics in Advanced/Metastatic Tumors.	Malignant Solid Tumor	11/1/2026
NCT02171104	Phase 2	Canadian Profiling and Targeted Agent Utilization Trial (CAPTUR)	Non-Hodgkin Lymphoma, Multiple Myeloma, Advanced Solid Tumors	1/31/2027
NCT02029001	Phase 2	Adapting Treatment to the Tumor Molecular Alterations for Patients with Advanced Solid Tumors	Malignant Solid Neoplasms	10/1/2026
NCT02029001	Phase 3	Molecular Profiling of Advanced Soft-tissue Sarcomas	Soft Tissue Sarcoma	10/1/2025
NCT02029001	Phase 1	A Phase I Trial of Simmitinib in Advanced Solid Tumors	Advanced Solid Tumor	3/31/2025

## 6 Innovations in TAM-focused therapeutic strategies

Oncological advancements are revealing the significant role that TAMs play in the TME. This has inspired innovative therapeutic strategies that harness the complex biology of TAMs. These strategies, enriched by emerging technologies, are carving fresh and promising avenues in the treatment of cancer. Particularly notable are efforts to reprogram TAMs for cancer immunotherapy (14, 114) and the application of single-cell technologies for precision interventions (115–118).

### 6.1 Reprogramming TAMs for cancer immunotherapy

TAMs possess a dual nature in cancer development, particularly in aggressive cancers like gastric adenocarcinoma. They can transition between tumor-resistant M1 macrophages, which exert anti-tumor functions, and tumor-promoting M2 macrophages. Recent evidence suggests that M2-type macrophages, similar to TAMs, serve as prognostic markers for gastric cancer (119). Instead of eliminating TAMs altogether, current therapeutic strategies aim to modify their behavior by converting pro-tumorigenic M2-like TAMs into anti-tumor M1-like forms (14). This approach retains TAMs’ essential roles in tissue repair and homeostasis while harnessing their potential in the fight against cancer. M2 macrophages, which resemble TAMs, are increasingly recognized as independent prognostic factors in gastric cancer (119, 120). The utilization of TAMs as prognostic tools in clinical settings is on the rise, particularly in the context of therapeutic reprogramming. Instead of complete TAM removal, current strategies focus on behavior modification, transitioning pro-tumorigenic M2-like TAMs into anti-tumor M1-like counterparts (7, 14). This strategy maintains the advantageous properties of TAMs in tissue repair and homeostasis while engaging them in the battle against cancer.

### 6.2 Utilizing single-cell technologies for precision interventions

TAMs play critical roles in a range of cancers, notably endometrial and breast types. The impact of cancer on the transcriptional landscapes of monocytes and macrophages suggests consequential effects on patient outcomes. For instance, breast TAMs present a signature linked to aggressive cancer subtypes, correlating with decreased disease-specific survival (121, 122). Discovering the interactions between TAMs and cancer cells has led to the identification of potential therapeutic targets like SIGLEC1 and CCL8 (46, 121, 123, 124).

In stage II colon cancer, the varying effectiveness of post-surgery chemotherapy highlights the significance of reliable biomarkers. Notably, TAM ratios, such as CD206/CD68, are emerging as pivotal prognostic and predictive metrics for postoperative chemotherapy (125, 126). These insights not only deepen our grasp of TAMs’ function in cancer but also accentuate the imperative for precision interventions.

The evolving clarity of TAM populations within the TME underscores the necessity for sophisticated interventions. Advanced tools, like single-cell RNA sequencing (scRNA-seq) enable the identification of distinct TAM subsets, revealing their specific molecular imprints and operational states (12, 116, 127, 128). By combining lineage tracing with scRNA-seq, researchers like Casanova-Acebes et al. (117) have elucidated TAM dynamics in specific cancer models, advocating for bespoke therapeutic approaches that target distinct TAM groups. This refined comprehension, powered by cutting-edge technologies, foreshadows individualized treatments that reconfigure the TME to hinder tumor growth.

## 7 The future of TAM research

TAMs, prominent actors within TME, exhibit both facilitative and inhibitive roles in cancer progression, making them intriguing therapeutic targets. As TAM-centered clinical trials advance, their potential to redefine cancer immunotherapy becomes increasingly evident.

### 7.1 Precision in TAM-targeted therapeutics

Given the diverse roles of TAMs, precision in therapy is imperative. Recent studies advocate for restricting TAM influx into the TME to counter their tumor-promoting functions (13). Additionally, efforts to direct TAM polarization toward the anti-tumor M1 phenotype are gaining traction, which may enhance the TME’s immune responsiveness. Emphasis is also on curbing TAM-induced immune suppression (129). As we continue to gain insight, the targeting of distinct TAM subsets emerges as a crucial strategy, warranting an integrative understanding of the TME (130). Such holistic endeavors might propel a new epoch in cancer care.

### 7.2 Innovations in non-invasive TAM monitoring

Advancing TAM research necessitates the standardization of isolation techniques. In this context, cutting-edge tools, such as single-cell analysis (116, 118), have emerged as powerful assets. Single-cell analysis not only allows for the isolation and study of individual TAMs but also provides a window into the intricate nuances of TAM phenotypes. Furthermore, it sheds light on the dynamic interactions between TAMs and the TME (15). This innovative approach has the potential to revolutionize our understanding of TAMs by uncovering their heterogeneity and unveiling the intricacies of their crosstalk within the TME. These advancements in non-invasive TAM monitoring hold promise for more targeted and effective therapeutic strategies in the fight against cancer.

### 7.3 Navigating clinical trial & their implications

Optimizing TAM-oriented therapies requires rigorous clinical trials to validate both their efficacy and safety. Such endeavors also spotlight potential biomarkers to ascertain the most suitable patient groups for these interventions. By understanding the intricate interactions of TAM-TME engagements, opportunities for combinatorial treatments emerge, elevating the standards of cancer therapy.

### 7.4 Reflections and horizons

Within the complex world of oncology, TAMs are at the forefront. Unraveling their role in tumor dynamics will inspire novel therapeutic ventures. With this momentum, the domain of TAM research has the promise to revolutionize cancer immunotherapy, a sentiment gaining traction among researchers (131).

## 8 Conclusion

TAMs, central to cancer dynamics, both facilitate and counteract tumor progression. Their intricate roles pose significant therapeutic challenges. Yet, the advent of clinical trials reveals potential means to regulate TAMs, mitigating their immune-suppressive effects. Integrating these TAM-focused strategies with current treatments may revolutionize cancer immunotherapy.

A paramount shift in TAM research demands standardized methodologies for consistent and precise results. Breakthroughs such as single-cell analyses grant a profound understanding of TAM functions and their complex TME interplay. Concurrently, efforts to devise drugs targeting adverse TAM subsets are intensifying. Through careful assessment and biomarker application, patient selection for optimal benefits becomes feasible.

In summary, investigating TAMs isn’t merely scholarly; it bears the promise of transforming cancer treatment paradigms. Embracing the nuances and opportunities in TAM research brings us a step closer to improved outcomes for cancer patients.

## Author contributions

SS: Conceptualization, Formal Analysis, Investigation, Methodology, Resources, Validation, Visualization, Writing – original draft, Writing – review & editing, Data curation. HM: Conceptualization, Data curation, Formal Analysis, Investigation, Methodology, Resources, Validation, Visualization, Writing – original draft, Writing – review & editing. WM: Conceptualization, Formal Analysis, Investigation, Methodology, Resources, Validation, Visualization, Writing – original draft, Writing – review & editing, Project administration, Software, Supervision.
