# Corrigendum: Unraveling the enigma of tumor-associated macrophages: challenges, innovations, and the path to therapeutic breakthroughs

**DOI:** 10.3389/fimmu.2025.1606194

**Published:** 2025-04-22

**Authors:** Shengwen Shao, Huilai Miao, Wenxue Ma

**Affiliations:** ^1^ Clinical Research Center, The Second Affiliated Hospital of Guangdong Medical University, Zhanjiang, Guangdong, China; ^2^ Department of Hepatobiliary Surgery, The Second Affiliated Hospital of Guangdong Medical University, Zhanjiang, Guangdong, China; ^3^ Department of Hepatobiliary Surgery, Liaobu Hospital of Dongguan City, Dongguan, Guangdong, China; ^4^ Department of Medicine, Moores Cancer Center, and Sanford Stem Cell Institute, University of California, San Diego, La Jolla, CA, United States

**Keywords:** tumor-associated macrophages (TAM), tumor microenvironment (TME), phenotypic diversity, regulatory signaling pathways, clinical trials, cancer immunotherapy

In the published article, there was an error. The labels for M1 and M2 macrophage subtypes were mistakenly reversed due to an oversight by the authors.

A correction has been made to *Section 2. The multifaced role of TAMs in the TME,* Paragraph 1. This sentence previously stated: “TAMs possess the ability to transition between pro-tumor (M1) and anti- tumor (M2) roles.”

The corrected sentence appears below: “TAMs possess the ability to transition between pro-tumor (M2) and anti-tumor (M1) roles.”

The authors apologize for this error and state that this does not change the scientific conclusions of the article in any way. The original article has been updated.

